# A study on movement characteristics and distribution law of dust particles in open-pit coal mine

**DOI:** 10.1038/s41598-021-94131-6

**Published:** 2021-07-19

**Authors:** Tong Wu, Zhuo Yang, Aiai Wang, Kai Zhang, Bo Wang

**Affiliations:** 1 China Coal Technology & Engineering Group Shenyang Engineering Company, Shenyang, Liaoning, China; 2 China Coal Technology & Engineering Group Chongqing Research Institute, Chongqing, China

**Keywords:** Environmental sciences, Space physics, Engineering

## Abstract

Previous simulation studies of dust particles movement behavior in open-pit coal mines only aimed at a single operation link, and the macro simulation is lacking. This study seeks to explore microscopic migration and macroscopic diffusion of dust particles in the mining area by numerical simulation method. The movement characteristics revealed, and the distribution law of the dust particles in a stable state with the corresponding migration paths in the mining area were obtained respectively. The results showed that the increase amplitude of dust particles diffusion velocity was inversely proportional to particle size, which was vital for dust pollution in the mine. And the larger the particle size in the mining pit was, the lower the escape rate of the dust particles was. Dust particles distributed over a wide range that were not limited by space height, and the distribution characteristics of dust particles at different heights were basically the same. Meanwhile, it is found that the dust distribution in the two places is relatively concentrated due to the circulation phenomenon of the mining pit and the west dump. And wind action would accelerate the moving dust particles to reach a stable distribution state.

## Introduction

With the continuous development of China's social economy, the requirements and standards for environmental quality of the whole society are gradually increased. Coal mine exploitation not only promotes the social and economic development, but also causes harm to the surrounding atmospheric environment. According to the investigation, the average dust concentration in the open-pit coal mines of township enterprises in 10 counties in 7 provinces of China is 5 ~ 43 times of the standard, of which 13% reached the standard value^1^. Most of new open-pit coal mines located in the west of China, with Mongolia and Xinjiang leading the way. These areas are mostly water-resource shortage areas, dry soil, lack of rainfall, some areas or sandstorms, in which open-pit mines are more likely to discharge dust during production^2^. And survey results show that at present, water sprinkling and traditional dust removers have been set for some dust-producing processes such as crushing and transplanting in the open-pit mine in this area, but the dedusting effect is unsatisfactory. The research on the law of dust accumulation, migration and diffusion is a necessary link in the development of green mining in open-pit coal mines, and has a certain guiding significance for dust suppression and removal.

The operation of open-pit coal mines produces a large amount of dust particles, among which fine particles such as PM_2.5_ are difficult to settle and easy to affect the air quality over a wide range as the air movement by hundreds of kilometers. Dust is a basic occupational hazard that can lead to silicosis and other diseases^3^. The rise of dust concentration in the mining pits performs negative effects not only on the environment and human health, but also on the production efficiency of the mine operation system^4^. The effective control of dust pollution is crucial to improving the environmental quality of the mining area near urban cities, and ensuring the health of operators.

In terms of the evaluation of particles motor behavior with environmental effect, many foreign scholars have been carrying out large-scale researches, such as the saltation, creep and suspension movement of sand particles, which laid a foundation for the theory study on migration behavior of dust particles in mining areas^5,6^.In terms of the application of numerical simulation models, most researchers at home and abroad applied models to explore the diffusion and migration process of urban atmospheric environmental pollutants at present^7^. Simulation studies on dust particles in open-pit coal mines are mainly focused on a single operation link^8,9^, and little about macroscopic diffusion and distribution of dust particles in the whole mining area^10^.

Based on the research foundation above, this paper process dynamic simulation of dust particles movement behavior in mining area, then determined the distribution of dust particles in the whole mining area innovatively. Finally, movement characteristics and distribution law of dust particles in open-pit coal mines were obtained through comprehensive analysis, which provided new ideas and basic references for dust control research in mining area.

## Materials and methods

### Methods

The modeling tool was the Fluent 13.0 numerical simulation software^11^, which could solve multiphase flow problems including particles, drops and bubbles in the fluid while calculating the transport equation of fluid flow. In this simulation, the dual/multi-fluid model (multi-phase flow model) and particle orbit model (discrete phase model) were respectively applied to tackle the problems.

In the process of numerical simulation, the air flow was taken as the background fluid, and the dust phase was regarded as the particles dispersed in the background fluid. Flow control equations were Navier–Stokes equations which applied to three-dimensional steady incompressible flow, and the k-ε two-range model was used for turbulent flow equations. In the model, only momentum transmission was considered, while heat transfer was ignored. The velocity field and pressure distribution of the gas on the working face was determined by mathematical equations. For the discrete phase, the orbits and the mass transfer of particles were determined by integrating the differential equation of forces in Lagrangian coordinates^12,13^.

### Simulation process

The research objective was an open-pit mine in Inner Mongolia, which was a typical large open-pit coal mine in China and adjacent to a city, making it a special value subject. Lignite was the main type of coal produced in this mining area. The annual average wind speed was 4.6 m/s and the maximum wind speed was 20.7 m/s. The annual wind direction was mainly northwest.

The entire mining area model was modeled by 1:1 ratio based on the contour map of the mining area, with the bottom elevation of 740 m and the horizontal road elevation around pithead of 900 m. And the x-direction length of the model was 8400 m, the y-direction length was 7350 m. To improve the computational speed and accuracy of the model, the basic model was simplified on the premise of ensuring the true shape of the mine pit and not affecting the dust diffusion and migration path. And the z-direction vertical height of 1500 m was selected to establish the flow field model of the mining area.

**Figure 1:**
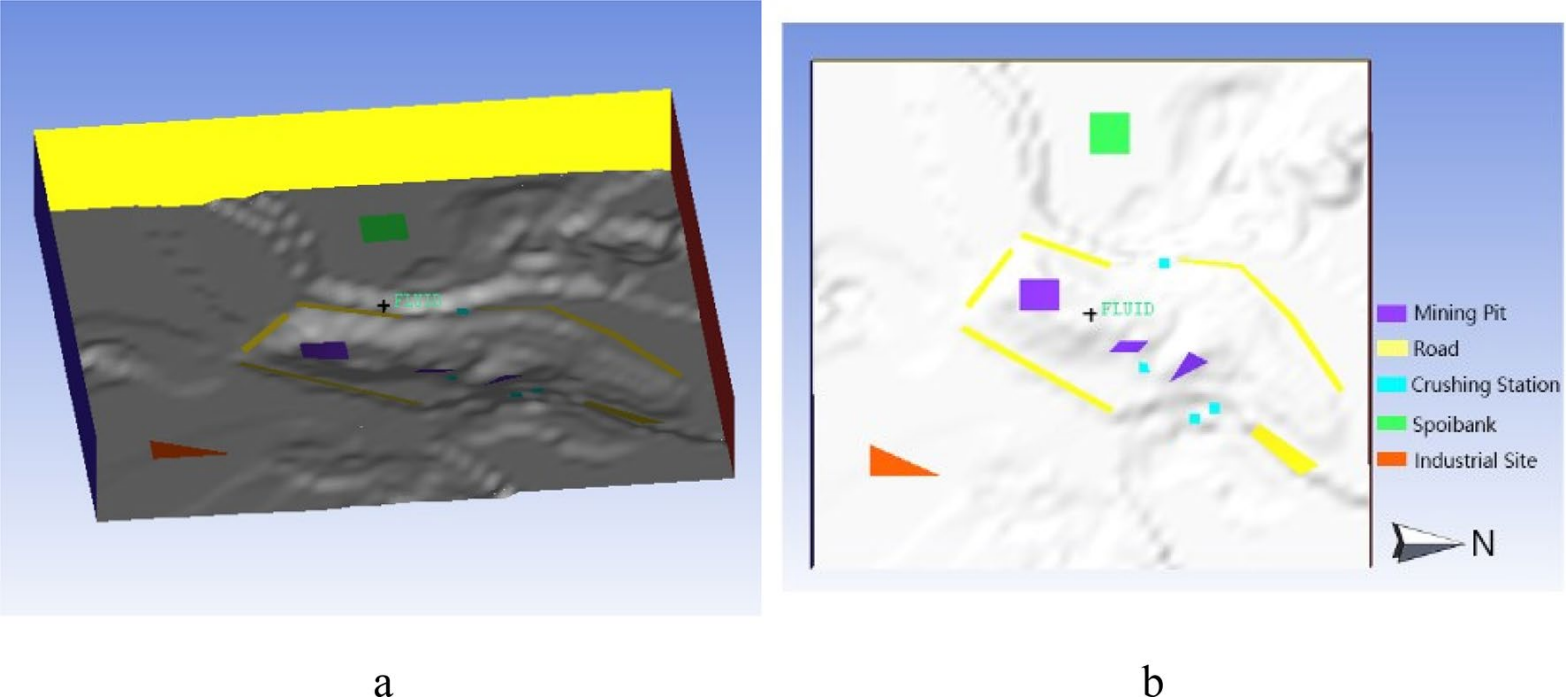
3D modeling diagram of mining area.

According to the operation condition and the emission intensity of each link, emission sources of the model was set: the three mining areas configured in the slope were set as three non-point emission sources; the west dump being used in the mining area was set as a non-point emission source; the four primary crushing stations in use were set as four non-point emission sources respectively. The industrial site was integrated into one non-point emission source to meet the calculation conditions of the model. And road dust was integrated into five non-point emission sources in combination with roads distribution and dust production in the mining area. The modelling results are shown in Fig. 1.

Grid division was carried out for the above model, in which the unit sizes were adjusted appropriately and the grid was manually encrypted at the emission sources. Then, the partitioned model was imported into the Fluent operation module for optimizing.

Considering the influence of wind force, two wind conditions of none and northwest wind with speed of 4.6 m/s were selected for the boundary conditions and dust production parameters setting of the model. The calculation parameters of the model are shown in Table 1.

**Table 1:** Calculation model parameters of migration and diffusion process.

Boundary conditions	Parameters setting
Turbulence model	k-epsilon standard
Gravitational acceleration /(m/s^2^)	9.8
Inlet boundary	Velocity-inlet
Outlet boundary	Outflow
Pressure–velocity coupling	SIMPLEC
Scheme of pressure interpolation	Standard
Spatial discretization	The second order wind
Discrete phase model	Open
Resistance characteristics	Spherical particles
Jet source type	Surface sources
Density of coal dust (kg/m^3^)	1500
Discrete term boundary	Trap

The simulation process was as follows: two-phase coupling solution was conducted for the dust phase and air phase. The airflow was set as incompressible fluid, and the dust phase was set as dust particles mixture in five sizes. The field flows performed as steady turbulence. To be balanced by iteration, the data file was derived and the dust diffusion distribution diagram was generated. Then, the dust was used as discrete phase particles for transient calculation, and the migration path of dust particles was derived after iterative equilibrium.

Furthermore, this paper simulated the micro-motion process of dust particles with five particle sizes and output their motion trajectories to explore the micro-motion characteristics of dust particles. In order to obtain the particle movement trajectory clearly, the mining pits of the mining area was chosen as simulation background.

**Figure 2:**
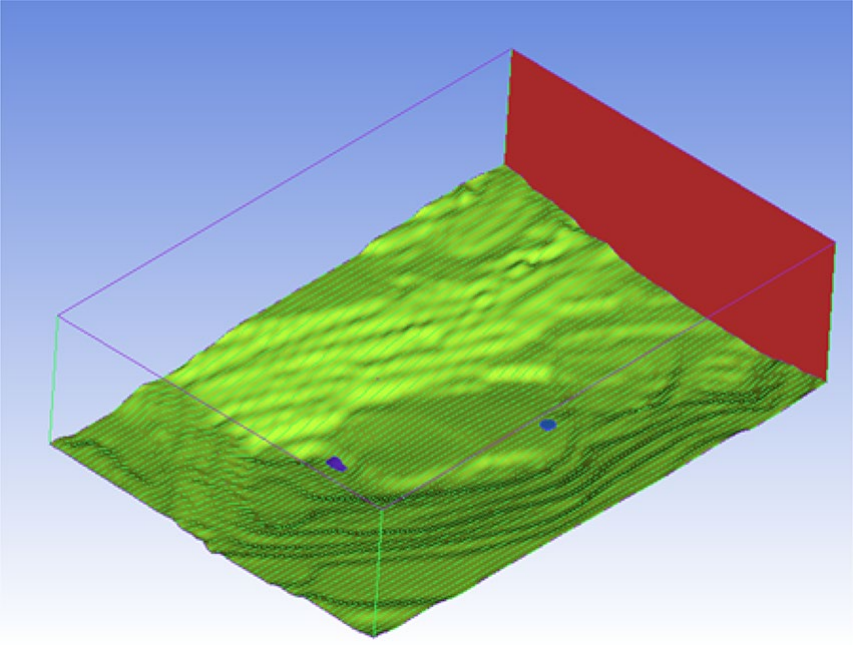
Modeling results of the mining pit flow field.

The mining pits area were modelled into a mining pit flow field model, which was established by x-direction length of 2627 m, Y-direction length of 1828 m, and the vertical height of z-direction of 1500 m. The modeling results of mining pits flow field were shown in Fig. 2 below.

Setting the wind condition as northwest and 4.6 m/s, and establishing an emission source at the bottom of the mining pit. The emission parameters of dust particles with particle sizes of 2.5 μm, 5 μm, 10 μm, 75 μm and 100 μm were set respectively to simulate the movement process of dust particles under the action of wind. The calculation parameters of the model are shown in Table 2.

**Table 2:** Calculation model parameters of micro-motion process.

Boundary conditions	Parameters setting
Turbulence model	k-epsilon Standard
Gravitational acceleration /(m/s^2^)	9.8
Inlet boundary	Velocity-inlet
Outlet boundary	outlet-vent
Pressure–velocity coupling	SIMPLE
Discrete phase model	Open
Resistance characteristics	Spherical particles
Jet source type	Surface sources
Density of coal dust (kg/m^3^)	1500
Discrete term boundary	Trap
wind speed	4.6 m/s
wind direction	North-west

### Validation of simulation

**Figure 3:**
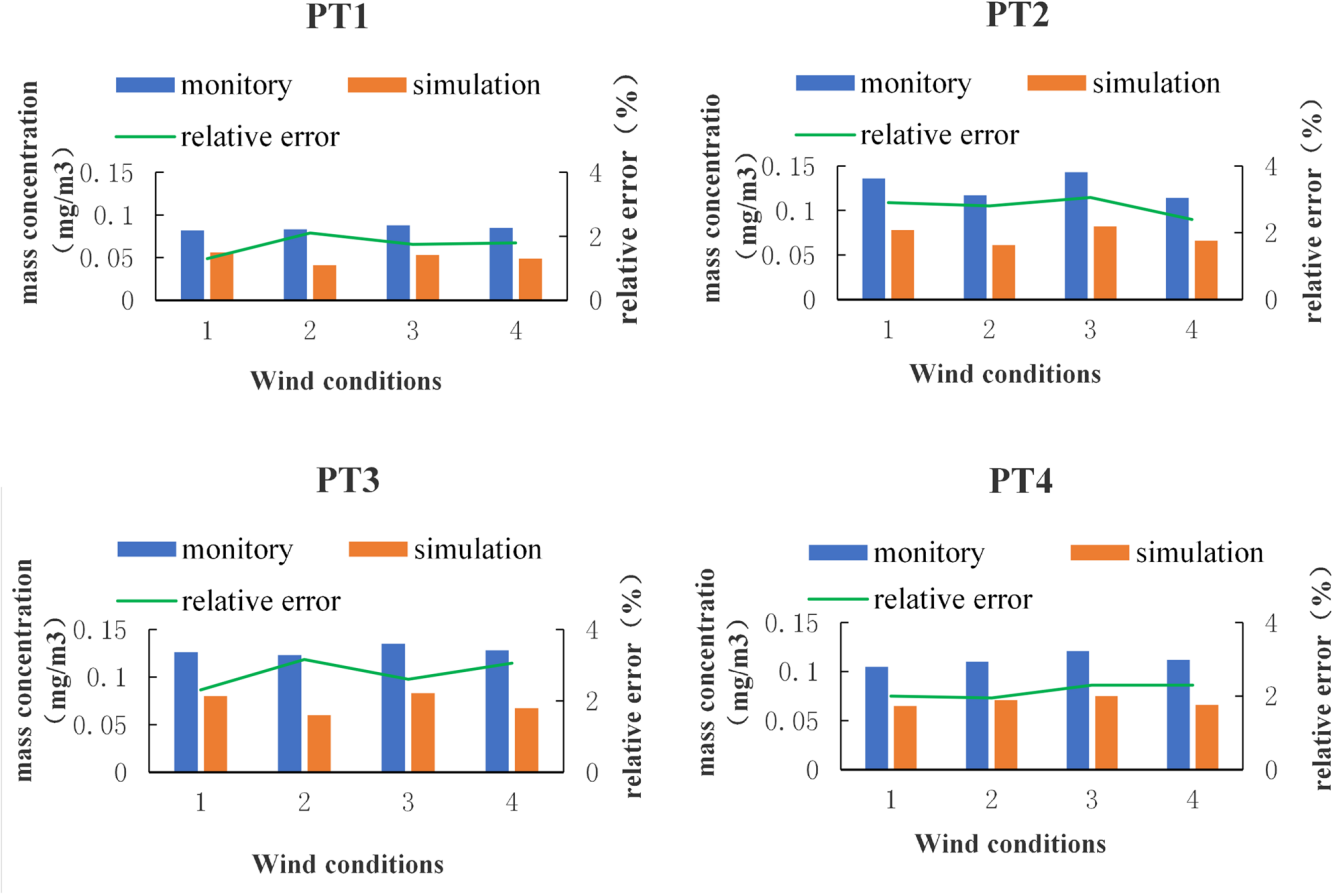
Comparison between simulated and monitored values.

To verify the rationality of modeling and parameters setting, simulating points were set referred to monitory points’ positions in the environmental report of this mine, and four different wind conditions in the environmental report were selected to synchronize in the simulation process. The mass concentration of four simulating points were output by model calculation and compared with monitored values, the comparison results were shown in Fig. 3.

It was found that the simulation results of all monitory points were slightly lower than the actual values in different degrees under the four wind conditions, which may be due to model optimization and emission sources’ integration in the modeling process. However, the relative error between the actual values and the simulated values was less than 5%, indicating that the simulation results were persuasive and both the process of modeling and parameter setting was relatively reasonable.

## Results

### Micro-motion simulation of particles

**Figure 4:**
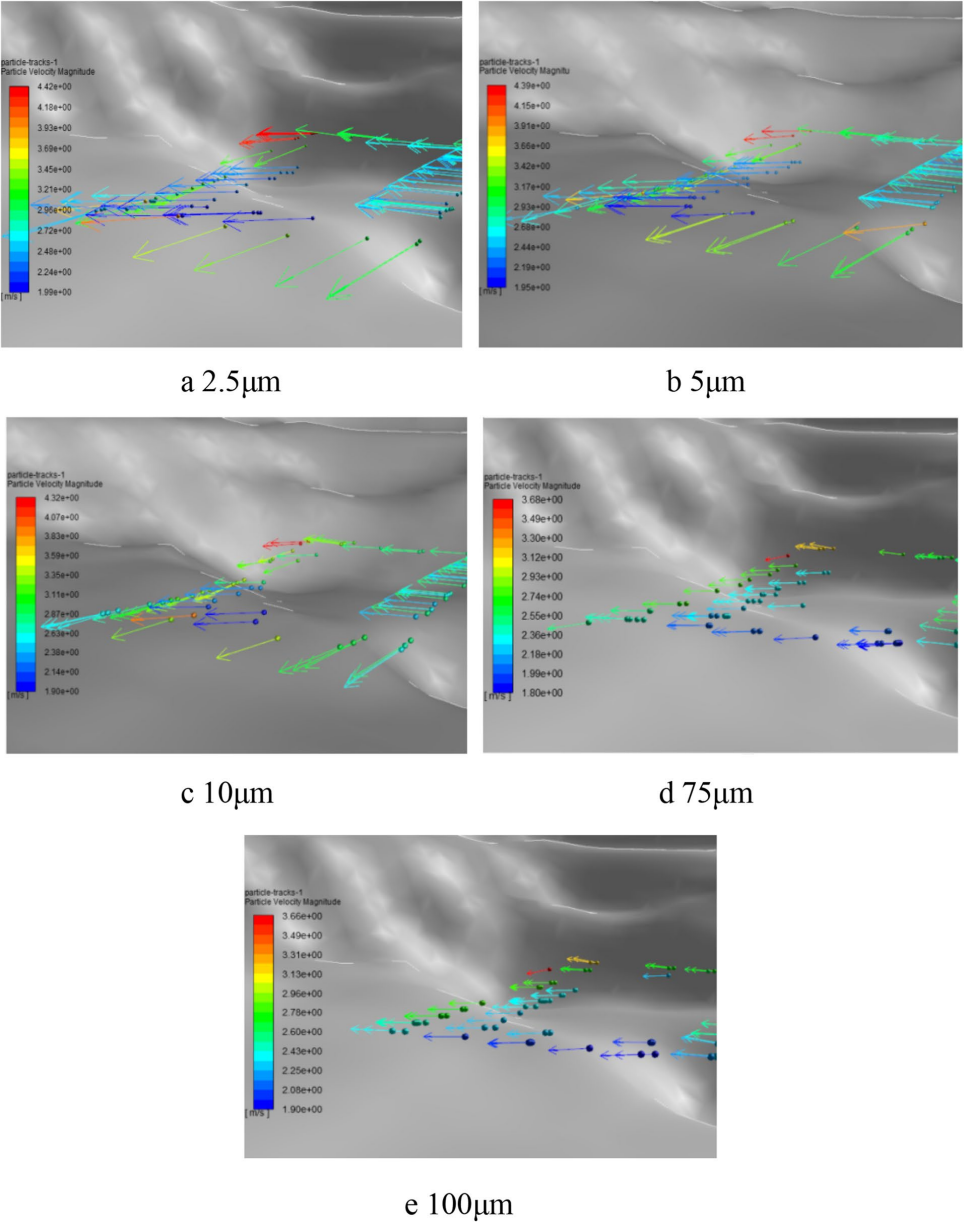
Movement trajectories of dust particles of five particle sizes by Fluent 13.0.

The simulation results showed that dust particles of five sizes reached a migration velocity of 4.5 m/s around 0.7 s and moved steadily along the wind direction at the migration velocity. The motion trajectories of dust particles of five sizes at 0.4 s were shown in Fig. 4 below.

Analysis of the Fig. 4 illustrated that dust particles of five particle sizes migrated in the same direction, and the length of movement path was inversely proportional to the particle size. At the same time, it was found that dust particles with particle size of 2.5 μm had the maximum movement velocity, and dust particles with particle size of 100 μm had the minimum movement velocity. The movement velocity is also inversely proportional to the dust particle size, which is close to the stable velocity of 4.5 m/s.

The simulation results above revealed motion characteristics of dust particles.

### Migration and distribution of particles

#### Windless condition

**Figure 5:**
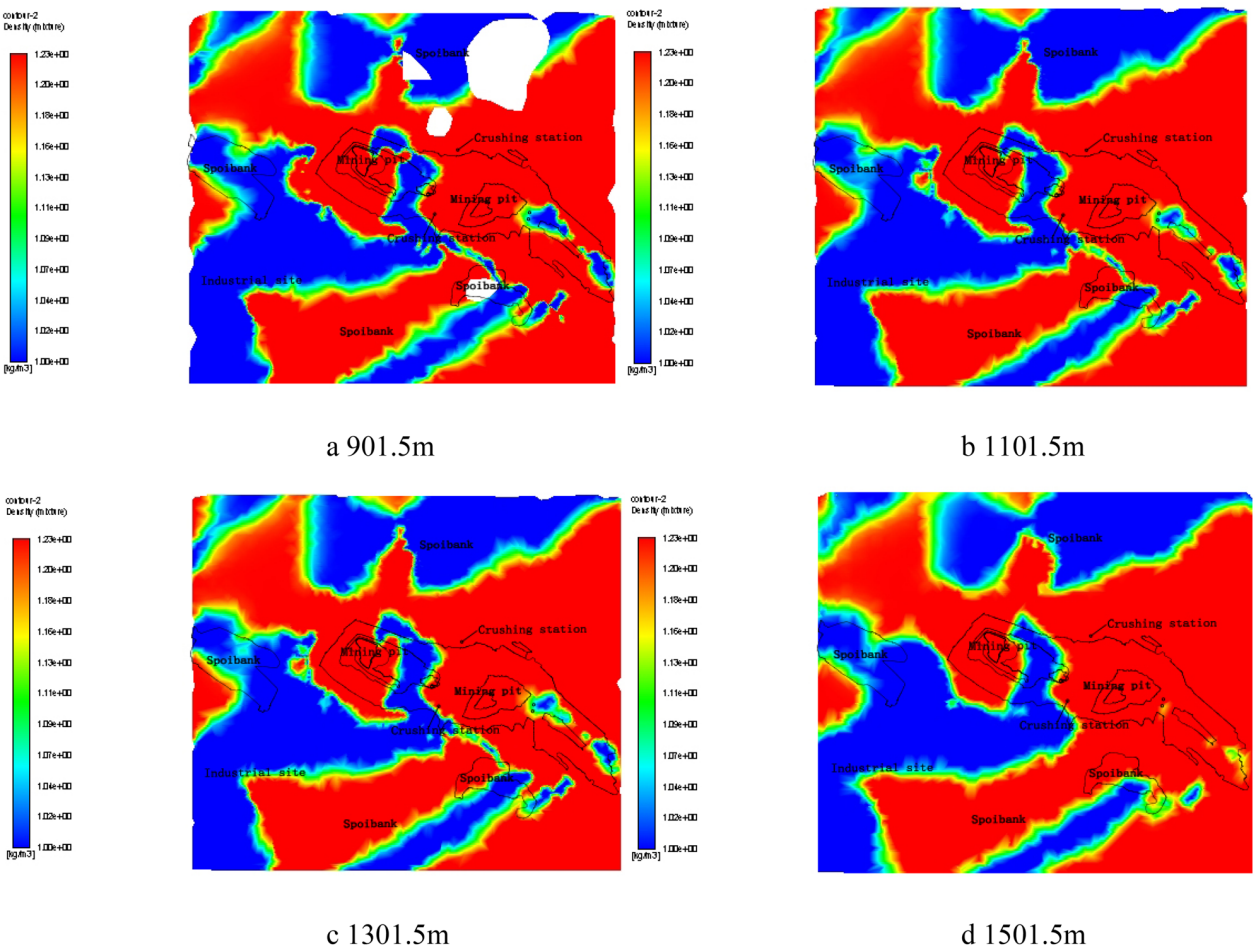
Dust distribution map under windless condition by Fluent 13.0.

Under the windless condition, the simulation results illustrated that dust particles in the mining area reach stable distribution after 5.5 h of diffusion and migration. We found that there was still dust at the top 1500 m of the model. And the final distribution of dust particles at different heights was described in Fig. 5 (The blue part is the dust distribution area, and the red part is the dust-free area).

Referring to the dust particles’ impact on mining operation and personnel health, four height planes were selected to illustrate the distribution of dust particles. They were the initial horizontal plane (Fig. 5a) at the vertical height of 1.5 m from the ground (the average height of human respiration) and the horizontal plane (Fig. 5b–d) upward for every 200 m later.

From the Fig. 5, the distribution range of dust particles was found to shrink gradually with height rising, which was related to the settling movement of dust particles. The rapid dust settling speed of large particles led to the small amount and sparse distribution of dust in the high space while the high number and wide distribution of dust in the low places. However, the distribution characteristics of dust particles at four heights were much the same, indicating that the diffusion range was wide-reaching and not easily restricted by the space height under the condition of no wind. That also reflected the hazard seriousness and control difficulty of dust pollution indirectly.

Combined with operation area partitioning of the mining area, an obvious accumulation of dust particles was found around the west dump and mining pit. Meanwhile, the dust particles whose distribution was relatively independent among each operation zone were found all gathered around the emission sources, which was related to the extensive area of the mine.

**Figure 6:**
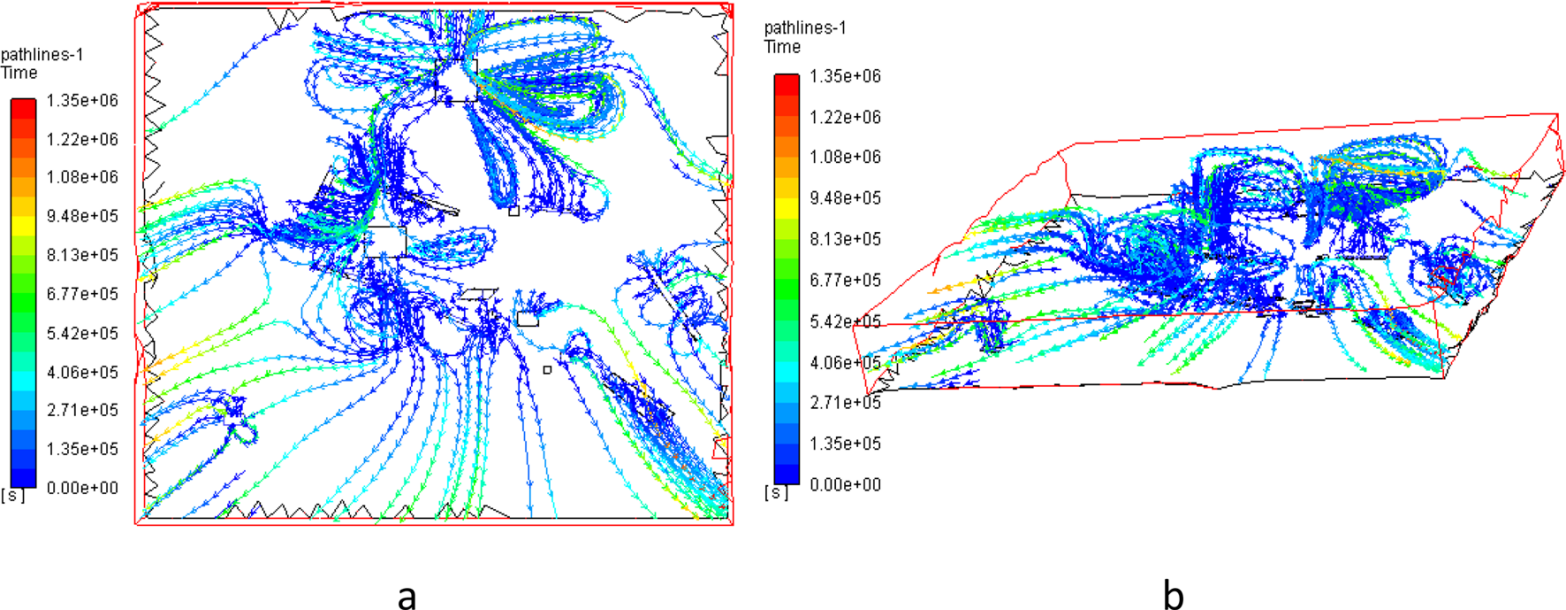
Dust particles migration path under windless wind by Fluent 13.0.

Based on the diffusion distribution results above, the dust particle migration path was solved and derived as shown in Fig. 6, where the color difference of the path corresponds to the migration time of dust particles on the path.

As can be seen from Fig. 6, dust particles stayed briefly in the center of the emission sources and then tended to diffuse outwards and stabilized by sides of emission sources.

At the same time, a circulation appeared in the complex terrain region (mining pit and west dump) flow field. The circulation was a large eddy formed by the re-circulation movement with dust particles migration that generated by air flow when it went through an open-pit coal mine slope and impacted by the geometry. It was also the reason for the obvious phenomenon that dust accumulated around the mining pit and the west dump^14,15^.

The circulation was mainly manifested as that wind currents arose in the slope opposite to the original wind direction. Combining the analysis of dust migration path variation near the mining pit, the vertical circulation appeared in the area. That is, the wind currents impinged on the windward slope in the southeast, then flowed to the lower windward slope and rose from the bottom of the mining pit to the upper leeward slope^16^.

The re-circulation can transport part of the airflow to the bottom of the mining pit and inject fresh air into the bottom of the pit. At the same time, dust particles will be easy to accumulate in the area around due to the existence of the re-circulation. To eliminate it, measures can be taken in the windward slope to cut down the reverse airflow flowing to the mining pit bottom so as to reduce the dust pollution in the area^17^.

#### Windy condition

**Figure 7:**
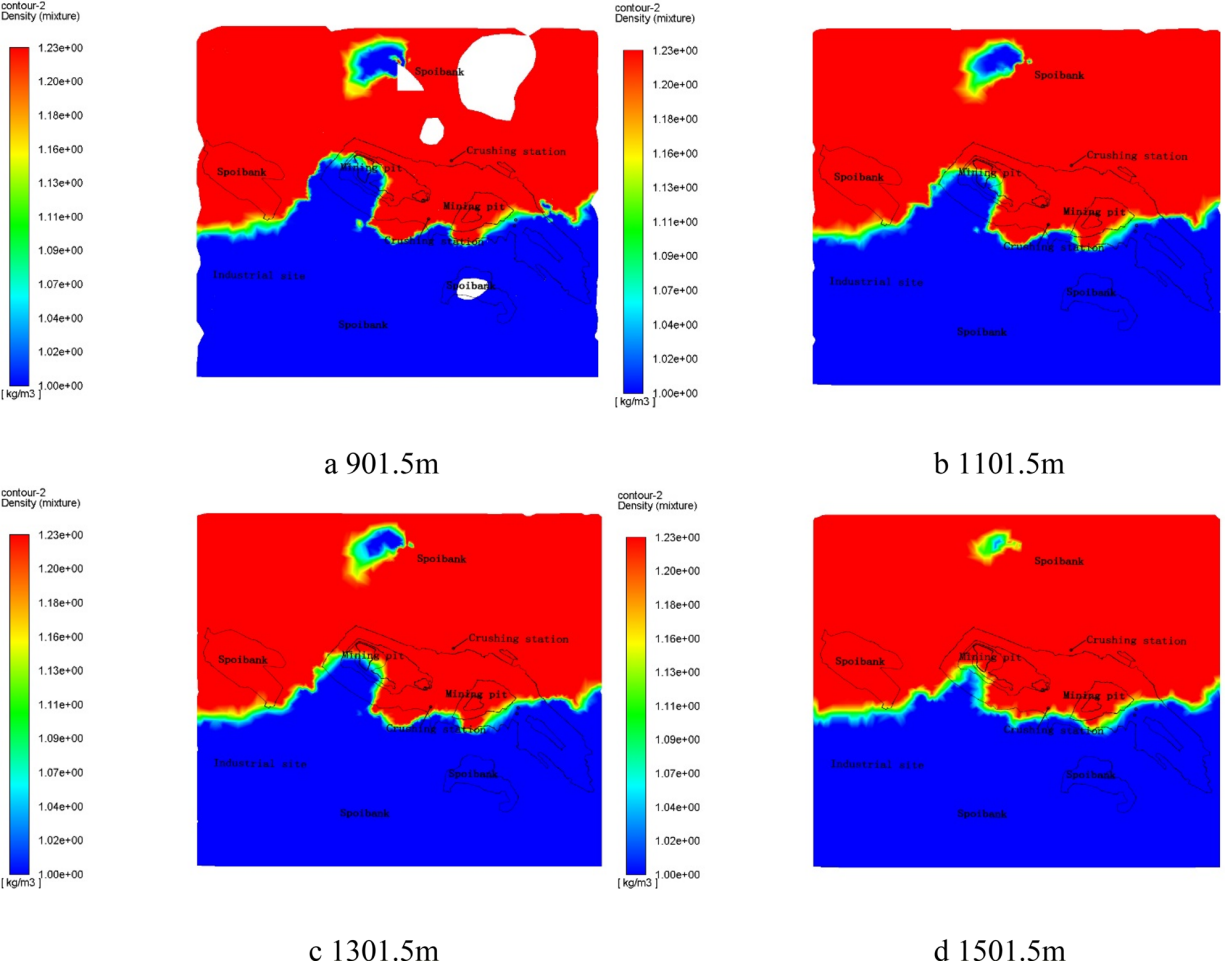
Dust distribution map under windy condition by Fluent 13.0.

Under the windy condition, the simulation results revealed that the dust particles in the mining area reached stable distribution after 1 h of diffusion and migration, which was speed up by wind. We found that there was still dust at the top 1,500 m of the model. And the final distribution of dust particles at different heights was described in Fig. 7 (The planes height was consistent with that of windless condition).

From the Fig. 7, the distribution range of dust particles shrunk gradually as height rose, and the distribution characteristics of dust particles at different heights were basically same under windy conditions. It indicated that the wind conditions played a decisive role in the migration direction of dust particles, and the impact of wind conditions on the migration and diffusion of dust particles was not limited by the space height. In addition, the emerge of the dust accumulation area in the east of the west dump may be related to the high abandonment height of the dump.

**Figure 8:**
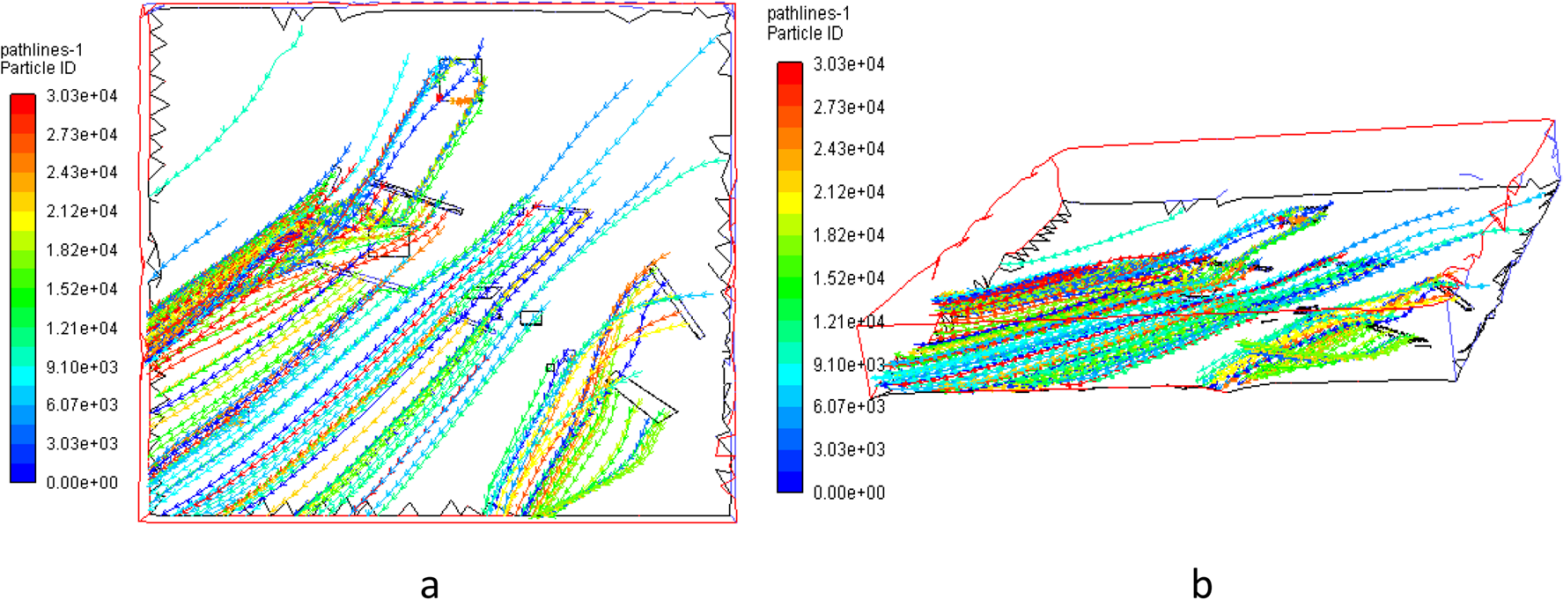
Dust particles migration path under windy condition by Fluent 13.0.

Based on the results above, the dust particles’ migration path under windy condition was determined, as showed in Fig. 8 below.

According to Fig. 8, the dust particles’ migration path performed obvious characteristics under windy condition, and all of them moved in the southeast direction along the wind flow.

Based on the analysis above, it could be derived that the migration and distribution law of dust particles in the open-pit mine area were the results of the comprehensive constraint of dust diffusion movement and wind flows^18,19^. It was suggested that wind power should be considered in the process of dust control.

### Escape rate of particles in mining pit

To delve into the dissipation law of dust particles in mining pit and quantify it, this study aimed at five particle sizes of dust particles (2.5 μm, 5 μm, 10 μm, 75 μm, 100 μm) and designed 24 groups of simulation experiments, including a total of eight wind direction settings as east, west, south and the north wind, and southeast to the northwest, northeast, southwest. Then three dust emission locations were selected as No. 1 (bottom of the pit), No. 2 (central north slope) and No.3 (pithead heap-leaching mining) respectively. And the dispersion of various particle sizes of dust particles in different wind conditions was explored.

The dust movement model was set as the discrete phase DPM model, the inlet boundary was set as the velocity inlet whose value was the average annual wind speed of 4.6 m/s, the outlet boundary was set as the pressure outlet, and the boundary condition of the wall (the inner of the pit) was set as the wall. In order to calculate the dust escape rate, the DPM type of inlet and outlet was set as Escape, and part of the wall type (the outer of the pit) was set as Trap. Assuming that the dust contacting walls would be captured by adsorption and considering the influence of turbulence on the dust particle movement, the simulation times of each experiment was set as 100 times to count the captured times of dust particles and calculate the escape rate of dust particles discharged from each dust source point under different wind directions. The statistical results were shown in Table 3 below.

**Table 3:** The dispersion rate of dust particles at each source point under different wind direction.

	No. 1 source point					No. 2 source point					No. 3 source point				
	2.5 μm	5 μm	10 μm	75 μm	100 μm	2.5 μm	5 μm	10 μm	75 μm	100 μm	2.5 μm	5 μm	10 μm	75 μm	100 μm
East	75%	73%	70%	1%	0	86%	85%	82%	2%	0	96%	95%	93%	5%	1%
West	55%	52%	51%	0	0	71%	70%	61%	0	0	81%	79%	75%	0	0
South	53%	51%	50%	0	0	70%	67%	62%	0	0	75%	75%	73%	0	0
North	72%	71%	71%	0	0	85%	83%	80%	1%	0	94%	94%	93%	3%	0
Southeast	64%	62%	61%	0	0	74%	71%	70%	0	0	88%	86%	82%	0	0
Northeast	74%	72%	71%	0	0	83%	82%	80%	1%	0	94%	93%	91%	2%	0
Southwest	47%	44%	43%	0	0	68%	62%	57%	0	0	50%	45%	40%	0	0
Northwest	63%	62%	59%	0	0	72%	70%	65%	0	0	85%	84%	80%	0	0

From the Table 3 above:

1. The dispersion degree of dust particles discharged from the same point was various under different wind direction. Generally speaking, the larger the particle size was, the lower the escape rate was.
2. The escape rate of dust particles from different source points was also inconsistent, and the principal manifestation was the dust particles discharged from the bottom of the mine have the lowest escape rate, and the pollution situation in this area is more serious than other places. It is recommended to add dust removal measures at the bottom of the mining pit.
3. In the range of 10 μm, there was a strong correlation between the dispersion rate of small size dust particles in mining pit, and dust particles tend to escape out of the mining pit. It is due to that settling movement of dust was affected by gravity and air resistance. Under specific wind conditions, small size dust particles will move by same trend.
4. The dispersion rate of large size particles above 75 μm was very low. And almost all of the particles settled down in the mining pit. Studies have shown that the settling velocity of dust particles increases significantly when the particle size is larger than 50 μm, which directly leads to the settling of large dust particles in the mining pit.

Then, affected by strong disturbance in the operation link of mining pit, large particles which have settled will be raised again, and the secondary dust will be formed. It is an important factor of visible dust pollution in the mining pit.

## Conclusions

This paper seeks to explore microscopic migration and macroscopic diffusion of dust particles in the mining area by numerical simulation method. The conclusions are as follows: (1) The increase amplitude of dust particles diffusion velocity was inversely proportional to particle size, which was vital for dust pollution phenomenon in the mine. (2) Dust particles distributed over a wide range that were not limited by space height, and the distribution characteristics of dust particles at different heights were basically the same. (3) Wind action would accelerate the moving dust particles to reach a stable distribution state. (4) The dust distribution in the two places is relatively concentrated due to the circulation phenomenon of the mining pit and the west dump. (5) In the mining pit, small size dust particles all escape out of the mining pit, and large size dust particles almost all settle down which are main pollution sources.

## Discussion

The results above revealed the migration characteristics and distribution law of dust particles in the mining area, and provided theoretical and technical reference for dust control research in open-pit coal mines.

Considering the movement characteristics uncovered by simulation results that large particle size dust particles tend to settle down in the mining pit. Under the condition of continuous operation, the large particles of dust increase constantly and can not be discharged to the outside of the pit. Thus correlative measures should be taken to clean up the large particles of dust and reduce pollution in the mining pit.

In addition, the simulation results confirm the key influence of wind field flow on dust migration process. In the dust-control process, combined with the auxiliary role of wind flow, higher dust suppression efficiency can be reached, such as increasing working time of producing more dust sources in windy weather appropriately, and decreasing working time of more dust sources in calm weather appropriately.
